# Developmental time course and effects of immunostressors that alter hormone-responsive behavior on microglia in the peripubertal and adult female mouse brain

**DOI:** 10.1371/journal.pone.0171381

**Published:** 2017-02-03

**Authors:** Mary K. Holder, Jeffrey D. Blaustein

**Affiliations:** 1 Department of Psychological and Brain Sciences, University of Massachusetts, Amherst, Massachusetts, United States of America; 2 Center for Neuroendocrine Studies, University of Massachusetts, Amherst, Massachusetts, United States of America; University of Modena and Reggio Emilia, ITALY

## Abstract

In female mice, the experience of being shipped from the breeder facility or a single injection of the bacterial endotoxin, lipopolysaccharide (LPS), during pubertal development alters the behavioral response to estradiol in adulthood as demonstrated by perturbations of estradiol’s effects on sexual behavior, cognitive function, as well as its anxiolytic and anti-depressive properties. Microglia, the primary type of immunocompetent cell within the brain, contribute to brain development and respond to stressors with marked and long-lasting morphological and functional changes. Here, we describe the morphology of microglia and their response to shipping and LPS in peripubertal and adult female mice. Peripubertal mice have more microglia with long, thick processes in the hippocampus, amygdala and hypothalamus as compared with adult mice in the absence of an immune challenge. An immune challenge also increases immunoreactivity (IR) of ionized calcium binding adaptor molecule 1 (Iba1), which is constitutively expressed in microglia. In the hippocampus, the age of animal was without effect on the increase in Iba1- IR following shipping from the breeder facility or LPS exposure. In the amygdala, we observed more Iba1-IR following shipping or LPS treatment in peripubertal mice, compared to adult mice. In the hypothalamus, there was a disassociation of the effects of shipping and LPS treatment as LPS treatment, but not shipping, induced an increase in Iba1-IR. Taken together these data indicate that microglial morphologies differ between pubertal and adult mice; moreover, the microglial response to complex stressors is greater in pubertal mice as compared to adult mice.

## Introduction

Puberty, the transition into a reproductively competent adult, and adolescence are developmental periods of great physiological, psychosocial, and cultural changes [1]. As such, it is also a time of considerable vulnerability to stressors. Stressful or traumatic events during the peripubertal period and adolescence contribute to the development and diagnosis of mental illness, such as anxiety or depression [2–6]. In rodents, the experience of a complex stressor, but not more commonly-used stressors such as restraint stress, food deprivation, or a multiple stressor regimen (restraint in combination with light exposure), during this period also increases stress reactivity, anxiety- and depression-like behaviors, and decreases cognitive performances in adulthood (reviewed in [7]). Female mice exposed to the stress of shipping or an immune challenge, lipopolysaccharide (LPS), during pubertal development demonstrate a reduction in hormone-induced sexual receptivity in adulthood [8–10].

In addition to inducing sexual receptivity, ovarian hormones, particularly estradiol, modulate the expression of anxiety- and depression-like behaviors; estradiol decreases both, the expression of anxiety- [11–14] and depression-like behaviors [15–17] in female rats and mice. Interestingly, although a combined treatment with estradiol and progesterone decreases anxiety-like behavior in ovariectomized (OVX) mice, treatment with LPS during the peripubertal period eliminates this [18]. Furthermore, rather than decreasing depression-like behaviors, estradiol treatment increased these behaviors in female mice treated with LPS during the peripubertal period [19]. These effects of LPS are eliminated if the treatment is delayed for two to four weeks.

The interaction between the neuroendocrine and immune systems has become a widely studied area in the development and mediation of mental illnesses. Microglia, the brain’s resident immune cells, play a critical role in brain development such as neurogenesis, migration, differentiation, synapse formation and neural plasticity [20–23]. Based on their role as the brain’s immune cells and in the normal neurodevelopmental processes, we postulate that microglia mediate the vulnerability of the pubertal brain to the effects of an immune challenge on long-terms changes in estradiol-regulated behaviors.

In support of this idea, a bacterial infection in male rat pups, but not juvenile male rats, leads to long-term microglial activation, increased brain cytokine levels, and behavioral changes in adulthood [24, 25]. Male rat pups also have an increased number of microglia at the same age, and female rats have more microglia in an activated phenotype as juveniles and adults than do males [26]. Females are more likely to be diagnosed with disorders that present during adolescence [27–30], suggesting that the developmental status of the microglia may underlie the onset of neuropsychiatric disorders.

Here, we describe the morphology of the microglia and their response to a complex stressor during and after pubertal development. We used ionized calcium binding adapter molecule 1 (Iba1) as a marker for microglia as it is uniformly distributed in the cytoplasm of microglia, making it suitable for analysis of microglia both in normal and in pathological conditions [31]. First, we characterized the morphology of microglia within the brains of peripubertal and adult female brains. Next, we examined the changes in microglia immunoreactivity following administration of LPS and the stress of shipping from a commercial breeding facility. These stressors were chosen because they result in enduring changes in behavioral responses to estradiol [8–10, 18, 19, 32].

## Material and methods

### Animals

Female C57Bl/6 or CD-1 mice were shipped from Charles River Laboratories (Kingston, NY, USA) at 3 weeks old and housed in an all female colony room under controlled temperature (24 ± 2°C) and reversed 14L:10D light cycle (lights off at 1000 h). Mice to be treated with LPS at 6 or 8 weeks old were shipped at 3 weeks of age, because they are insensitive to the long-term effects of shipping on estradiol-influenced behaviors at this age [9]. Mice were also shipped at 6 or 8 weeks of age to determine the effects of shipping at those ages. Shipping or LPS administration at 6 weeks of age causes the biggest alteration in hormone-responsive behaviors; therefore, this age was chosen to examine the effects of the pubertal stressors on microglia. Mice were housed in groups of four in clear polycarbonate cages with *ad libitum* access to food (Teklad 2014, phytoestrogen-reduced diet, Harlan Laboratories, Madison, WI) and water in glass bottles. This study was approved by the University of Massachusetts, Amherst Institutional Animal Care and Use Committee (Protocol # 2010–0073, 2013–0081) and performed in accordance with the National Institutes of Health Guide for Care and Use of Laboratory Animals. No animals used in these experiments became ill or died prior to the experimental endpoint. All efforts were made to minimize animal suffering and to reduce the number of animals used. Experimenters blinded as to treatment groups of animals and brain sections conducted all experiments.

### LPS treatment

LPS from *E*. *coli* serotype O26:B6 was obtained from Sigma Aldrich (St. Louis, MO, USA). The LPS was dissolved in sterile saline vehicle to a concentration of 0.1mg/ml. Mice were randomly assigned to treatment groups and received a single intraperitoneal injection of LPS (1.5mg/kg body weight) or an equivalent volume of sterile saline vehicle and returned to their home cage immediately following injection. The dose of LPS when administered during the peripubertal period has previously been demonstrated to decrease behavioral responses to estradiol in adulthood [8, 10, 18, 19, 32]. Consistent with previous reports [8, 10, 18, 19, 32], this dose of LPS produced moderate sickness behavior.

### Sickness behavior

Sickness behavior was scored at 30 min, 4 h, and 24 h following injections of LPS or saline or arrival from the breeder facility by two observers who were blind to the treatment conditions. Sickness behavior was scored using a 0–4 scale modified from [33, 34]. Mice were assessed for lethargy as defined by decreased locomotion, huddling as defined by a curled body posture, ptosis as defined by drooping or closed eyelids, and piloerection.

### Tissue collection

Mice were deeply anesthetized with pentobarbital (100mg/kg), and brains were removed and submersed into a fixative solution of 4% paraformaldehyde in phosphate buffered saline (PBS; 0.05M, pH 7.4) at 4°C, followed by cryoprotection in 30% sucrose in PBS. After cryoprotection, the brains were frozen on dry ice and stored at -80°C until processed for immunocytochemistry. Brains were sectioned (30μm) in the coronal plane in a cryostat and stored in a cryoprotectant solution (ethylene glycol/sucrose in sodium phosphate buffer) until immunostained.

### Immunocytochemistry

Cohorts containing free-floating sections from all treatment groups were rinsed in 0.05M Tris-buffered saline (TBS; pH 7.2). Sections were then rinsed in a solution containing 10% saline, 1% gelatin, 0.2% sodium azide, and 0.2% Triton-X in 0.05M TBS (gel TBS). Sections were then incubated in a blocking buffer containing 2% normal goat serum, 1% bovine serum albumin, and 3.5% hydrogen peroxide in gel TBS for 1 h. Sections were then incubated for 48h at 4°C with a rabbit polyclonal antibody raised against ionized calcium binding adapter molecule 1 (Iba1; Wako, Cat. #019–19741, RRID:AB_839504) at a dilution of 1:10,000 in 2% normal goat serum and 0.5% Triton X-100 in gel TBS. After primary incubation, the sections were rinsed in TBS and incubated in biotinylated secondary antibody (goat anti-rabbit; Vector Laboratories Cat. # BA-1000, RRID:AB_2313606) in 2% normal goat serum in gel TBS for 90 min, followed by washes in gel TBS. Finally, the sections were incubated with an avidin-biotin horseradish peroxidase complex (Vectastain ABC, Elite Kit; Vector Laboratories Cat. # PK-7100, RRID:AB_2336827) for 90 min at room temperature, washed in gel TBS and then washed in TBS. The sections were visualized with nickel sulfate and 3,3’-diaminobenzidine tetrahydrochloride (DAB kit; Vector Laboratories Cat. # SK-4100, RRID:AB_2336382). After visualization, the sections were rinsed in TBS, mounted serially onto gelatin-coated glass slides, and coverslipped.

#### Unbiased stereology

Anatomically matched sections containing the arcuate nucleus, ventromedial hypothalamus (VMH), basolateral amygdala (BLA), and hippocampus were analyzed. Both sides of the nuclei were included in the analysis. Iba1 labeled cells were counted using the optical fractionator method within StereoInvestigator software (Microbrightfield Inc., Williston, VT, USA). For analysis, we set an optical dissector height of 10μm with a 1-μm guard zone on top and bottom, and counted stained cells within each frame. Cells were only counted as positive if the entire cell body was visible and the stain appeared uniformly dark throughout the cell.

A 75 μm by 75 μm counting frame was used to count cells throughout each section for each section counted. For each animal, we analyzed every fourth section throughout the brain regions and analyzed the three representative sections in each brain region. The counting contour of each region matched Plates 42, 43, and 44 of the mouse brain atlas [35]. For each section examined, the area was calculated by the StereoInvestigator software and was based upon the boundaries of the contour tracings. Volume estimates were obtained by summing the areas given by the Cavalieri estimator, and them multiplying the sum of these areas with the pre-histology thickness of each sample by the number of sections examined Table 1. The volumes did not differ between peripubertal and post-pubertal animals for any of the regions counted.

**Table 1:** Average volumes for each brain region compared across age.

Region	Avg. volume (μm^3^) ± SEM Peripubertal	Avg. volume (μm^3^) ± SEM Adult	Statistics
BLA	216.458 x 10^6^ ± 6.963 x 10^6^	236.802 x 10^6^ ± 8.629 x 10^6^	t_(6)_ = 1.835; p = 0.12
DG	75.424 x 10^6^ ± 5.423 x 10^6^	67.858 x 10^6^ ± 3.551 x 10^6^	t_(6)_ = 1.167; p = 0.29
CA1	147.172 x 10^6^ ± 16.403 x 10^6^	155.356 x 10^6^ ± 23.783 x 10^6^	t_(6)_ = 0.283; p = 0.79
CA3	228.393 x 10^6^ ± 11.856 x 10^6^	25.301 x 10^6^ ± 11.112 x 10^6^	t_(6)_ = 1.519; p = 0.18
ARC	58.994 x 10^6^ ± 1.479 x 10^6^	66.159 x 10^6^ ± 4.837 x 10^6^	t_(6)_ = 1.416; p = 0.21
VMH	233.554 x 10^6^ ± 5.467 x 10^6^	229.935 x 10^6^ ± 9.084 x 10^6^	t_(6)_ = 0.341; p = 0.74

The mean volume ± SEM for each brain region is listed along with the statistical analysis for this data. We found that age of the animals was without effect on the region volumes. ARC, arcuate nucleus; BLA, Basolateral Amygdala; CA1, CA1 region of the hippocampus; CA3, CA3 region of hippocampus; DG, dentate gyrus; VMH, ventromedial hypothalamus.

Iba1-positive cells were classified into four morphological types based on their cell shape and configuration of their processes based upon the classification criteria described elsewhere [26]. These four types consisted of round/amoeboid microglia, microglia with stout processes, microglia with thicker longer processes, and microglia with thinner, more ramified processes. The total number of each classification of Iba1-positive cells across all representative sections for each brain region was counted. The total numbers of cells for all sections were analyzed using a mixed ANOVA with the within subjects factor of morphology and between subjects factor of age.

#### Thresholding image analysis

Sections containing the arcuate, VMH, BLA, and hippocampus were analyzed. Both sides of the nuclei were included in the analysis. One best-matched section for each brain area for each mouse was imaged using a Nikon Optiphot-2 microscope connected to a QImaging Micropublisher RTV 5.0 digital color camera (Surrey, BC, Canada). Gray-scale images of the arcuate, VMH, BLA, and hippocampus were analyzed with Image J 1.43 (National Institutes of Health). The regions of analysis were outlined bilaterally in each section, and cross-sectional area was measured. The Iba1-immunoreactivity (IR) was quantified for each nucleus using a maximum entropy thresholding algorithm [36] with threshold set to capture only clearly labeled cell nuclei and processes. Ramified microglia have been demonstrated to transition into reactive microglia, characterized by shorter and thicker processes and expanded cell bodies, leading to an increase in the area of microglia [37, 38]. Therefore, Iba1-IR was quantified by the mean area stained [39, 40].

### Statistical analysis

Data are represented as means + SEM or ± SEM where appropriate. For experiment 1, a mixed ANOVA (within subjects for morphology, between subjects for age) was used, followed by Fishers’ LSD post hoc comparisons. A two-tailed t test was used to compare the total numbers of microglia counted for each region. For experiment 2, two-way ANOVAs with age and peripubertal treatment as independent measure, followed by Fishers’ LSD post hoc comparisons were used. All statistical tests were conducted using the IBM Statistics SPSS (Chicago, IL) on a Macintosh Duo-core computer.

### Experiment 1: Which microglial morphological phenotypes are displayed during the peripubertal period and in adulthood?

**Fig 1:**
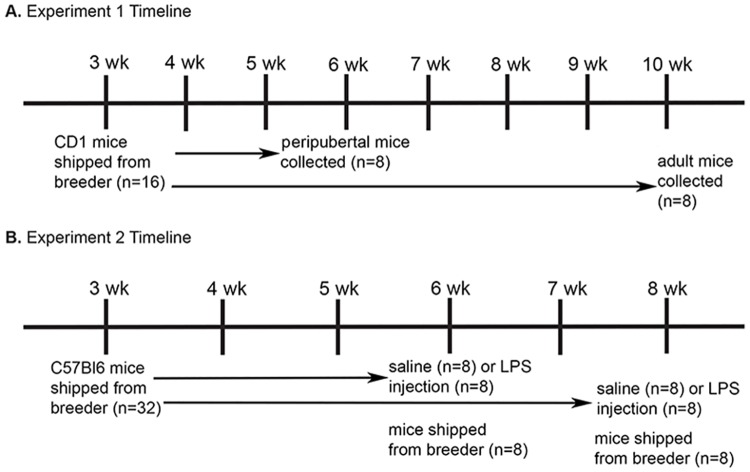
Experimental timelines. (A) CD1 mice were shipped from the breeder facility at 3 weeks old, and brains were collected at 6 or 10 weeks old. Following collection, brains were immunoprocessed for Iba1-IR. (B) C57Bl/6 mice were shipped from the breeder facility at 3 weeks old. Mice were injected with LPS or saline vehicle at 6 or 8 weeks old. In addition, mice were shipped from the breeder facility at either 6 or 8 weeks old. Twenty-four hours following the injections or arrival from the breeder facility, mice were collected, and their brains were immunoprocessed for Iba1-IR.

Sixteen female CD1 mice, shipped at three weeks of age, were euthanized, and brains were removed from the skull at six (peripubertal) or ten (adult) weeks of age (Fig 1). These time points were chosen because LPS treatment at six weeks causes reduction in hormone-induced sexual receptivity in adulthood [10], alters estradiol’s anxiolytic [18], anti-depressive [19], and pro-cognitive properties [32]. In contrast, LPS treatment at 10 weeks does not alter these behavioral measures. Following submersion fixation, brains were cyroprotected, and processed for Iba1-IR.

### Experiment 2: Do immunostressores (e.g., shipping or LPS) capable of altering hormone-responsive behavior in adulthood increase activation of microglia during the peripubertal period?

In order to determine if shipping from the breeder facility or LPS treatment induced an increased response in the microglia of peripubertal mice, thirty-two female C57Bl/6 mice were shipped at three weeks old, six weeks old (n = 8) or eight weeks old (n = 8). The mice shipped at three weeks old were divided into two groups: those injected with either saline (n = 8) or LPS (n = 8) at 6 weeks old, and those injected with saline (n = 8) or LPS (n = 8) at 8 weeks old (Fig 1). Twenty-four after LPS injection or arrival from the breeder facility, mice were euthanized and brains were removed from the skull. Following submersion fixations, brains were cyroprotected, and processed for Iba1-IR.

## Results

### Experiment 1: What are the microglial morphological phenotypes displayed during the peripubertal period and in adulthood?

#### Amygdala

**Fig 2:**
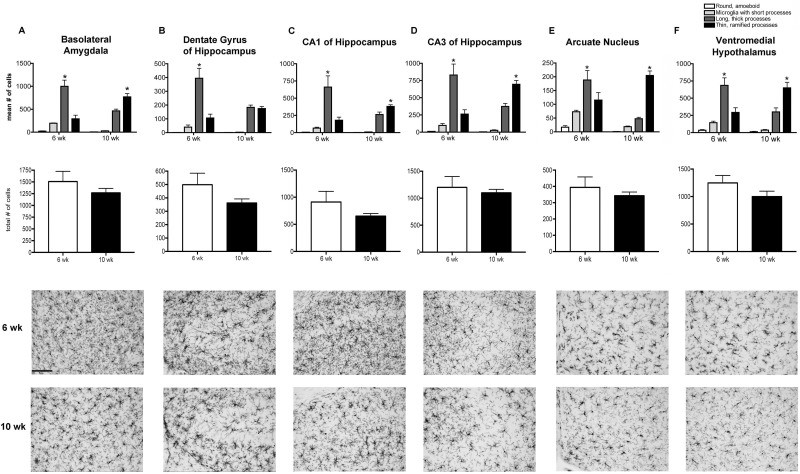
Microglia morphology is significantly affected by age in the brain regions analyzed. Within the (A) basolateral amygdala; (B) dentate gyrus, (C) CA1, and (D) CA3 of the hippocampus; (E) arcuate nucleus; and (F) ventromedial nucleus of the hypothalamus analysis revealed a significant interaction of age and microglial morphology as peripubertal (6 wk) females had significantly more microglia with thick, long processes than postpubertal (10 wk) females (*p<0.05). In addition, adult females also had more ramified microglial with thin processes in the basolateral amygdala, CA1 and CA3 of the hippocampus, the arcuate nucleus, and the ventromedial nucleus of the hypothalamus than peripubertal females (*p<0.05). There were no differences in the total numbers of microglia counted in any region. Data represent the mean + SEM of all Iba1-immunopositive cells in each morphological category across all sections analyzed.

In the amygdala, there was a significant interaction of the age of the animal and the microglial morphology [F_(3,24)_ = 22.15, p<0.0001] (Fig 2). In 6 wk old mice, more microglia had long, thick processes (p<0.001) compared to all other morphologies. In contrast, the long, thin ramified microglia predominate in the 10 wk old mice (p<0.001). There was no difference in the total numbers of microglia counted [t_(6)_ = 1.01, p = 0.35].

#### Hippocampus

There was a significant interaction of the age of the animal and the microglial morphology in both the dentate gyrus [F_(3,24)_ = 8.255, p<0.001] (Fig 2) and CA1 [F_(3,24)_ = 7.919, p<0.001] (Fig 2) of the hippocampus. In both of these regions, the 6 wk old mice have significantly more microglia with long, thick processes than those with the round/amoeboid morphology (p<0.0001), microglia with short processes (p <0.0001), or microglia with long thin ramified processes (p <0.0001). While more microglia display the long, thin morphology compared to round/amoeboid microglia (p<0.001) or those with short processes (p< 0.001) in 10 wk mice, the number of microglia with long, thick processes did not differ from those displaying long, thin processes (dentate gyrus p = 0.83; CA1 p = 0.20). There were no differences in the total numbers of microglia counted in the dentate gyrus [t_(6)_ = 1.501, p = 0.18] or the CA1 [t_(6)_ = 1.29, p = 0.24].

There was also a significant interaction of the age of the animal and the microglial morphology in the CA3 of the hippocampus [F_(3,24)_ = 14.83, p<0.0001] (Fig 2). In the CA3, there were more microglia with long, thick processes compared to any other morphology (p<0.0001) in 6 wk old mice. In contrast, the long, thin ramified microglia predominate in the 10 wk old mice (CA3 p<0.001). There was no differences in the total numbers of microglia counted in the CA3 [t_(6)_ = 0.47, p = 0.65].

#### Hypothalamus

There was a significant interaction of the age of the animal and the microglial morphology in both the arcuate nucleus [F_(3,24)_ = 15.72, p<0.0001] (Fig 2) and the VMH [F_(3,24)_ = 13.56, p<0.0001] (Fig 2). In both of these regions, there were more microglia with long, thick processes compared to those that were round/amoeboid (arcuate p<0.0001; VMH p<0.0001), with short processes (arcuate p<0.0001; VMH p<0.0001), or with long, thin processes (arcuate p<0.001; VMH p<0.0001) in 6 wk old mice. In contrast, the long, thin ramified microglia predominate in the 10 wk old mice (arcuate p<0.0001; VMH p<0.001 compared to all other morphologies). Importantly, there was no difference in the total numbers of microglia counted in the arcuate nucleus [t_(6)_ = 0.74, p = 0.49] or VMH [t_(6)_ = 1.489, p = 0.19].

### Experiment 2: Do immunostressores (e.g., shipping or LPS) capable of altering hormone-responsive behavior in adulthood increase activation of microglia during the peripubertal period?

#### Sickness behavior

**Fig 3:**
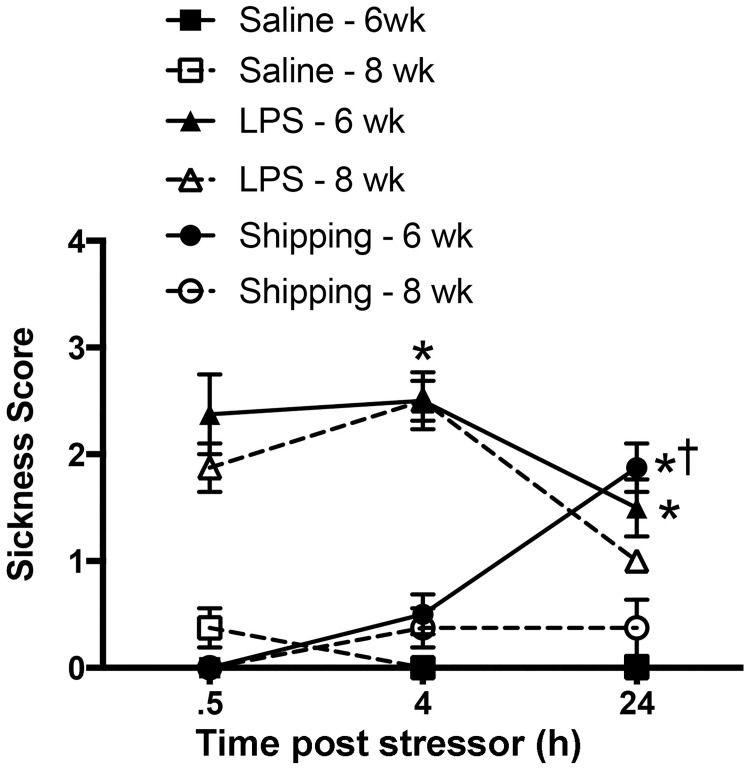
Sickness scores. Sickness scores in C57Bl/6 peripubertal (6 wk) or postpubertal (8 wk) mice shipped from the breeder facility or injected with either saline or LPS at 6 or 8 wk of age. Significantly greater than saline controls (*p<0.05). Significantly greater than 8 wk, within the same treatment (^†^p<0.05). Data represent mean sickness scores ± SEM.

There was a significant interaction of time, age, and the treatment on the sickness behaviors as defined by decreased locomotion (lethargy), huddling, ptosis, and piloerection displayed by the mice [F_(4, 84)_ = 3.333 p<0.05] (Fig 3). There was also a significant interaction of age and treatment on sickness behaviors [F_(2,84)_ = 3.408, p<0.05] (Fig 3). Post hoc analysis indicates that mice shipped at 6 wk old or had a significant increase in sickness behavior 24 h after arrival at the animal facility compared to age-matched, saline-treated mice (p<0.05) and mice shipped at 8 wk old (p<0.05), suggesting that shipping may have been more stressful during pubertal development. LPS treatment caused a significant increase in both 6- and 8-week-old mice at all time points examined compared to the respective saline controls (p<0.001). There were no differences in the sickness behavior displayed by 6 or 8 week-old mice following LPS administration.

#### Amygdala

**Fig 4:**
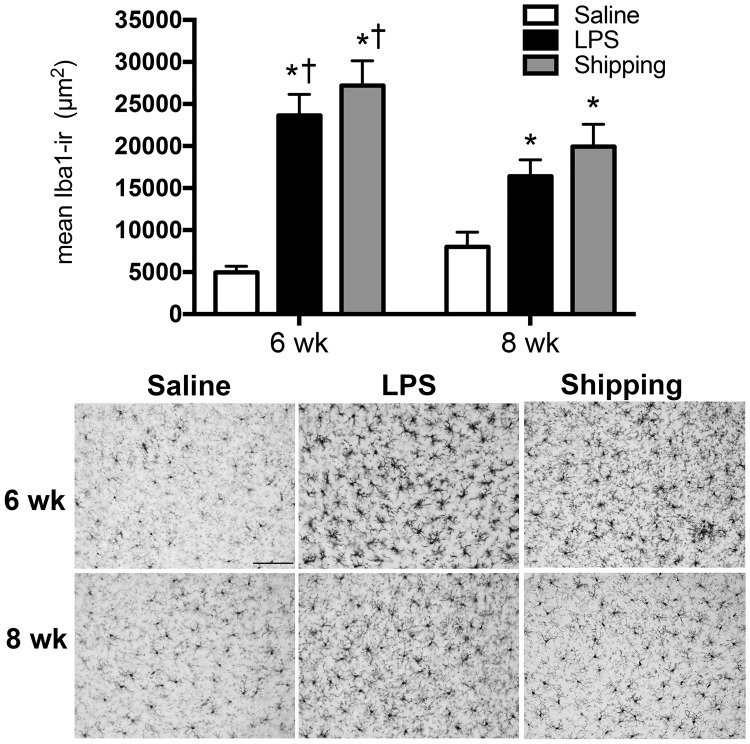
Iba1 immuoreactivity in basolateral amygdala following shipping or injections of either saline or LPS in peripubertal (6 wk) and postpubertal (8 wk) C57Bl/6 mice. Shipping from the breeder facility and LPS injection increases Iba1 immunoreactivity in the basolateral amygdala in both 6 wk and 8 wk mice, compared to age-matched saline-treated controls (*p<0.05). Shipping and LPS treatment induced a greater increase in Iba1-immunoreactivity in the arcuate nucleus of 6 wk mice, compared to 8 wk mice within the same treatment (^†^p<0.05).

There was a significant interaction of age and treatment on the Iba-IR [F_(2,41)_ = 3.41, p<0.05] (Fig 4). Both LPS treatment and shipping from the breeder facility caused an increase in the area of Iba1 immunostain in both 6 week (p<0.0001 for both LPS and shipping) and 8 week-old (LPS p<0.05; shipping p<0.01) female mice as compared with saline-treated mice. Importantly, the Iba-IR induced following either LPS treatment or shipping was greater in 6 week-old mice as compared to 8 week-old mice (p<0.05 for both LPS and shipping).

#### Hippocampus

**Fig 5:**
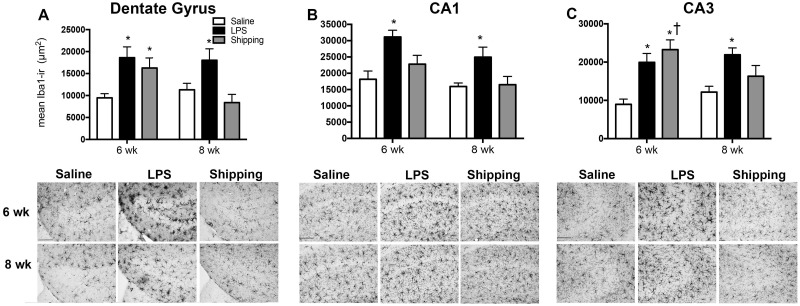
Iba1 immuoreactivity in hippocampus following shipping or injections of either saline or LPS in peripubertal (6 wk) and postpubertal (8 wk) C57Bl/6 mice. Treatment with LPS increases Iba1 immunoreactivity (IR) in the (A) dentate gyrus, (B) CA1 and (C) CA3 of the hippocampus in both 6 wk and 8 wk mice, compared to age-matched saline-treated controls (*p<0.05). Shipping from the breeder facility also increases Iba-IR in the dentate gyrus and CA3 in 6 wk mice, compared to saline-treated controls (*p<0.05). Significantly greater than 8 wk, within the same treatment (^†^p<0.05). Data represent mean Iba1-immunoreactivity + SEM.

There was no interaction of treatment type and age on the Iba1-IR in the dentate gyrus of the hippocampus [F_(2,42)_ = 0.76, p = 0.47] (Fig 5). There was, however, a significant main effect of treatment on the area of Iba1 immunostain [F_(2,42)_ = 17.77, p<0.001] (Fig 5). Post hoc tests indicate that LPS treatment increased the area of Iba1 stain in both 6 week- (p<0.001) and 8 week-old (p<0.001) female mice compared to respective saline-controls. In addition, shipping from the breeder facility increased Iba1-IR in 6 (p<0.05), but not 8 week-old mice, compared to saline-treated mice.

There was no interaction of the treatment type or age of treatment on Iba1-IR in the CA1 of the hippocampus [F_(2,42)_ = 0.942, p = 0.41] (Fig 5). There were a significant main effects of treatment [F_(2,42)_ = 15.93, p<0.0001] (Fig 5) and age [F_(1,42)_ = 9,458, p<0.01] (Fig 5). Post hoc tests indicate that LPS treatment increased Iba1-IR in both 6 week- (p<0.001) and 8 week-old (p<0.001) female mice compared to respective saline-controls. In addition, Iba1-IR did not differ in mice shipped at 6 weeks from the saline-treated mice (p = 0.18) nor from the mice shipped at 8 weeks (p = 0.85). There were, however, trends for LPS administration (p = 0.08) or shipping (p = 0.07) from the breeder facility to increase Iba1-IR in 6 week-old mice, compared to 8 week-old mice.

There was a significant interaction of treatment and age on area of Iba1 stained in the CA3 of the hippocampus [F_(2,42)_ = 3.375, p<0.05] (Fig 5). Post hoc tests indicate that LPS treatment increased the area of Iba1-IR in both 6 week- (p<0.001) and 8 week-old (p<0.001) female mice compared to respective saline-controls. In addition, shipping at 6 weeks from the breeder facility also increased Iba1-IR compared to saline-treated controls (p<0.05). While there was no difference in Iba1-IR in mice treated with LPS at 6 compared with 8 wks (p = 0.51), mice shipped at 6 weeks-old had increased Iba1-IR compared to mice shipped at 8 weeks-old (p<0.05).

#### Hypothalamus

**Fig 6:**
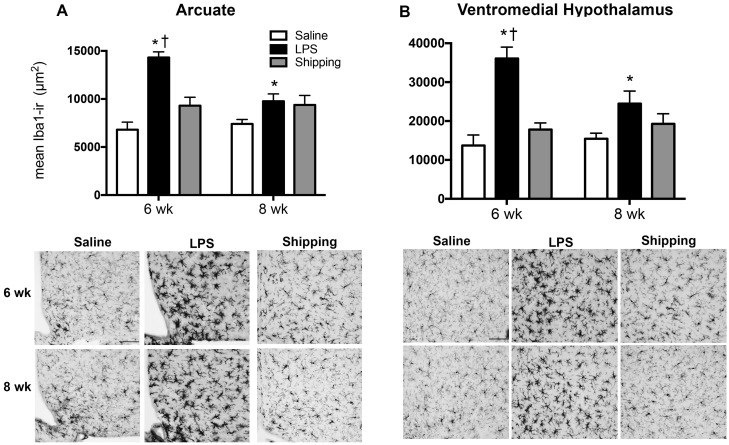
Iba1 Immunoreactivity in the arcuate nucleus and ventromedial hypothalamus following shipping or injections of either saline or LPS in peripubertal (6 wk) and postpubertal (8 wk) C57Bl/6 mice. Treatment of LPS increases the optical density of Iba1-immunostrain and the Iba1 immunoreactivity in the (A) arcuate (B) ventromedial hypothalamus 6 wk mice, compared to age-matched saline-treated controls (*p<0.05). In the arcuate nucleus, LPS treatment also increased Iba-immunoreactivity in 8 wk mice, compared to saline-treated controls. LPS treatment induced a greater increase in Iba1-immunoreactivity in the arcuate nucleus of 6 wk mice, compared to 8 wk mice treated with LPS (^†^p<0.05). Data represent Iba1-immunoreactivity + SEM.

There was a significant interaction of both age and pubertal treatment on the Iba1-IR in the arcuate nucleus [F_(2,40)_ = 7.258, p<0.01] (Fig 6). Post hoc tests indicate that in the arcuate nucleus, LPS treatment significantly increased the Iba1-IR in both 6 week (p<0.001) and 8-week-old mice (p<0.05), as compared to saline-treated controls. Importantly, LPS treatment at 6 weeks causes a significantly greater increase in Iba1-IR, compared to LPS treatment at 8 weeks (p<0.05). Shipping from the breeder facility did not increase Iba1-IR in 6 week-old mice (p = 0.50) and trended toward significant in 8 week-old mice (p = 0.28), compared to controls.

There was a significant interaction of both age and pubertal treatment on the mean area of Iba1-stain in the VMH [F_(2,40)_ = 4.728, p<0.05] (Fig 6). As with the arcuate nucleus, LPS treatment significantly increased the area of Iba1-stain in 6 week (p<0.001) and 8-week-old mice (p<0.05), as compared to saline-treated controls. LPS treatment at 6 weeks causes a significantly greater increase in Iba1-IR, compared to LPS treatment at 8 weeks (p<0.01). Shipping did not increase Iba-IR in 6 (p = 0.26) or 8 week-old mice (p = 0.28) compared to controls.

## Discussion

The present study sought to examine the morphological phenotypes of microglia and the response to shipping from the breeder facility or and LPS during pubertal development and in adulthood. These stressors were chosen because they, but not other typical stressors, have enduring influences on behavioral response to hormones in adulthood [8–10, 18, 19, 32]. In general, the microglia have longer, thicker processes during the peripubertal period as compared to a more thinly ramified phenotype post-puberty in the amygdala, CA1 and CA3 of the hippocampus, arcuate and VMH Table 2. Following shipping from the commercial breeder facility or an injection of LPS, there was a greater increase in Iba1-immunoreactivity in peripubertal mice than in adult mice Table 2. Taken together, these studies support the hypothesis that developmental status and morphology of the microglia contribute to the vulnerability of stressors experienced peripubertally.

**Table 2:** Summary findings.

	Summary Findings	
Brain Region	Experiment 1	Experiment 2
BLA	More microglial with thick, long processes in peripubertal mice More microglia with thin, ramified processes in adult mice	Increased Iba1-IR following LPS treatment or shipping in peripubertal and adult mice Greater increase in Iba1-IR in peripubertal mice following LPS treatment or shipping
DG	More microglial with thick, long processes in peripubertal mice	Increased Iba1-IR following LPS treatment in pubertal and adult mice Increased Iba1-IR following shipping in peripubertal mice
CA1	More microglial with thick, long processes in peripubertal mice More microglia with thin, ramified processes in adult mice	Increased Iba1-IR following LPS treatment in peripubertal and adult mice
CA3	More microglial with thick, long processes in peripubertal mice More microglia with thin, ramified processes in adult mice	Increased Iba1-IR following LPS treatment in peripubertal and adult mice Increased Iba1-IR in mice shipped during the peripubertal period
ARC	More microglial with thick, long processes in peripubertal mice More microglia with thin, ramified processes in adult mice	Increased Iba1-IR following LPS treatment in peripubertal and adult mice Greater increase in Iba1-IR in peripubertal mice following LPS treatment
VMH	More microglial with thick, long processes in peripubertal mice More microglia with thin, ramified processes in adult mice	Increased Iba1-IR following LPS treatment in peripubertal and adult mice Greater increase in Iba1-IR in peripubertal mice following LPS treatment

ARC, arcuate nucleus; BLA, Basolateral Amygdala; CA1, CA1 region of the hippocampus; CA3, CA3 region of hippocampus; DG, dentate gyrus; VMH, ventromedial hypothalamus.

Microglia have important roles in brain development: synaptic refinement [41–46], as ongoing cell genesis, synaptogenesis, and cell death occurs throughout pubertal development and contribute to the expression of adult behaviors [1, 47–49]. Therefore, it seems likely that the different morphologies of microglia [26] in the BLA, hippocampus and hypothalamus of pubertal mice may reflect the contribution of microglia to the ongoing neural development, which is largely completed post-pubertally, except in areas like the dentate gyrus of the hippocampus. Indeed, in the dentate gyrus, the microglia have long, thick processes during and after puberty.

It was expected that shipment from the commercial breeding facility and the administration of LPS would lead to similar increases in Iba1-IR, as both of these experiences induce similar alterations in behavioral response to estradiol in adulthood [8–10]. However, shipping and LPS both increase Iba1-IR during the peripubertal period than post-pubertally only in the basolateral amygdala. A primary function of the basolateral amygdala is the stimulation of fear and anxiety [50]. It is tempting to speculate that the perturbations of anxiety behaviors following peripubertal LPS result from altered trajectories of the ongoing neurodevelopment. That is, the increased activation could result in increases in cell death or an altered trajectory of synaptic pruning or refinement.

The experience of shipping and LPS both lead to altered sexual behavior; therefore, an increase in Iba1-IR was expected in VMH and arcuate nucleus, as both of these regions are involved in hormone-induced, female sexual behavior [51–55] and have decreased expression of ERα following shipping during the vulnerable period [10]. While LPS administration increase Iba1-IR in pubertal, compared to post-pubertal mice, shipping was without effect on Iba1-IR in these areas. The discordance between behavioral effects of LPS (e.g., alterations in cognitive and anxiety- and depression-like behaviors [18, 19, 32]) and the differential increase in Iba1-IR extends to the hippocampus, in which age was a factor in response to shipping, but did not influence the amount of microglial activation LPS administration.

It is also important to note that we measured sickness behavior 4 and 24 h after the arrival of the animals. The time after the onset of the stress of shipping (e.g., removal from home cage at breeder, packing, transit from the breeder facility, etc.) is not known. It is possible that the critical time to observe the effects shipping was missed. Perhaps examining the microglial activation from the onset of shipping, as opposed to arrival in the laboratory would be more comparable to 4 and 24 h after the LPS injection. It is also important to note that animals that were shipped from the breeder facility did not receive a vehicle i.p. injection; therefore, it is possible that some of effects on sickness behavior and/or microglial activation are due to the experience of the i.p. injection. Furthermore, perhaps some of the behavioral changes (e.g., sexual and cognitive behaviors) may be secondary to alterations in fear and anxiety.

Region-specific microglial profiles have been identified based upon the gene expression of proteins involved in not only in activation, but also in pathogen recognition or phagocytosis [56]. One source of the region-specific microglial activation could be the response to ovarian hormones, particularly estradiol. The presence of the ovary, and estradiol in particular, has been reported to be necessary for LPS to trigger an inflammatory response in the microglia [57]. Brain regions such as the arcuate nucleus and the VMH have high concentration of ERα; in contrast, the hippocampus has a high concentration of ERβ [58]. It is likely that the age differences in microglial activation following a stressor may result from these differences in ER populations, as the receptors have been reported to share antagonistic, sequential, or synergistic relationships [59]. The mice used in the current study were ovarian intact, as were the mice used in the previous studies, further supporting the idea that the signaling mechanisms of ERs may contribute to activation of microglia. It should be noted that these animals were not staged for ovarian cyclicity or hormone-controlled. This is, in part, because the mice used in the previous studies [8–10, 18, 19] were also not staged or hormonally manipulated. However, the contributions of the different ERs and the role of the ovarian hormones on the microglial activation or gene expression or protein profiles of the microglia, particularly in the peripubertal period, remain to be determined.

Taken together, the data presented here suggest that the peripubertal microglia may be more responsive to the experiences of shipping or LPS, which then lead to increases both Iba1-IR and sickness behavior in peripubertal mice. Although not directly tested, these data also are consistent with the hypothesis that increased inflammation underlies the peripubertal-stressor-induced perturbations in behavioral responses to estradiol and progesterone in adulthood. Furthermore, these data also yield insight as to why the peripubertal period may be a critical time for the development of mental disorders.

## References

[1] Sisk CL, Foster DL. The neural basis of puberty and adolescence. Nat Neurosci. 2004;7(10):1040–7. Epub 2004/09/29. 10.1038/nn1326 15452575

[2] Ge X, Conger RD, Elder GH Jr. Pubertal transition, stressful life events, and the emergence of gender differences in adolescent depressive symptoms. Dev Psychol. 2001;37(3):404–17. Epub 2001/05/24. 11370915 10.1037//0012-1649.37.3.404

[3] Grant KE, Compas BE, Stuhlmacher AF, Thurm AE, McMahon SD, Halpert JA. Stressors and child and adolescent psychopathology: moving from markers to mechanisms of risk. Psychol Bull. 2003;129(3):447–66. Epub 2003/06/06. 12784938 10.1037/0033-2909.129.3.447

[4] Grant KE, Compas BE, Thurm AE, McMahon SD, Gipson PY. Stressors and child and adolescent psychopathology: measurement issues and prospective effects. J Clin Child Adolesc Psychol. 2004;33(2):412–25. Epub 2004/05/12. 10.1207/s15374424jccp3302_23 15136206

[5] Silverman AB, Reinherz HZ, Giaconia RM. The long-term sequelae of child and adolescent abuse: a longitudinal community study. Child Abuse Negl. 1996;20(8):709–23. Epub 1996/08/01. 8866117 10.1016/0145-2134(96)00059-2

[6] Turner RJ, Lloyd DA. Stress burden and the lifetime incidence of psychiatric disorder in young adults: racial and ethnic contrasts. Arch Gen Psychiatry. 2004;61(5):481–8. Epub 2004/05/05. 10.1001/archpsyc.61.5.481 15123493

[7] Holder MK, Blaustein JD. Puberty and adolescence as a time of vulnerability to stressors that alter neurobehavioral processes. Front Neuroendocrinol. 2014;35(1):89–110. Epub 2013/11/05. 10.1016/j.yfrne.2013.10.004 24184692 PMC3946873

[8] Laroche J, Gasbarro L, Herman JP, Blaustein JD. Enduring influences of peripubertal/adolescent stressors on behavioral response to estradiol and progesterone in adult female mice. Endocrinology. 2009;150(8):3717–25. Epub 2009/04/18. 10.1210/en.2009-0099 19372198 PMC2717861

[9] Laroche J, Gasbarro L, Herman JP, Blaustein JD. Reduced behavioral response to gonadal hormones in mice shipped during the peripubertal/adolescent period. Endocrinology. 2009;150(5):2351–8. Epub 2009/01/10. 10.1210/en.2008-1595 19131570 PMC2671909

[10] Ismail N, Garas P, Blaustein JD. Long-term effects of pubertal stressors on female sexual receptivity and estrogen receptor-alpha expression in CD-1 female mice. Horm Behav. 2011;59(4):565–71. Epub 2011/03/08. 10.1016/j.yhbeh.2011.02.010 21376052 PMC3085923

[11] Diaz-Veliz G, Soto V, Dussaubat N, Mora S. Influence of the estrous cycle, ovariectomy and estradiol replacement upon the acquisition of conditioned avoidance responses in rats. Physiol Behav. 1989;46(3):397–401. Epub 1989/09/01. 2623060 10.1016/0031-9384(89)90010-3

[12] Diaz-Veliz G, Urresta F, Dussaubat N, Mora S. Effects of estradiol replacement in ovariectomized rats on conditioned avoidance responses and other behaviors. Physiol Behav. 1991;50(1):61–5. Epub 1991/07/01. 1946732 10.1016/0031-9384(91)90498-d

[13] Diaz-Veliz G, Alarcon T, Espinoza C, Dussaubat N, Mora S. Ketanserin and anxiety levels: influence of gender, estrous cycle, ovariectomy and ovarian hormones in female rats. Pharmacol Biochem Behav. 1997;58(3):637–42. Epub 1997/11/05. 9329052 10.1016/s0091-3057(97)90004-6

[14] Galeeva A, Tuohimaa P. Analysis of mouse plus-maze behavior modulated by ovarian steroids. Behav Brain Res. 2001;119(1):41–7. Epub 2001/02/13. 11164524 10.1016/s0166-4328(00)00341-7

[15] Okada M, Hayashi N, Kometani M, Nakao K, Inukai T. Influences of ovariectomy and continuous replacement of 17beta-estradiol on the tail skin temperature and behavior in the forced swimming test in rats. Jpn J Pharmacol. 1997;73(1):93–6. Epub 1997/01/01. 9032138 10.1254/jjp.73.93

[16] Rachman IM, Unnerstall JR, Pfaff DW, Cohen RS. Estrogen alters behavior and forebrain c-fos expression in ovariectomized rats subjected to the forced swim test. Proc Natl Acad Sci U S A. 1998;95(23):13941–6. Epub 1998/11/13. 9811905 10.1073/pnas.95.23.13941 PMC24977

[17] Dalla C, Antoniou K, Papadopoulou-Daifoti Z, Balthazart J, Bakker J. Oestrogen-deficient female aromatase knockout (ArKO) mice exhibit depressive-like symptomatology. Eur J Neurosci. 2004;20(1):217–28. Epub 2004/07/13. 10.1111/j.1460-9568.2004.03443.x 15245494

[18] Olesen KM, Ismail N, Merchasin ED, Blaustein JD. Long-term alteration of anxiolytic effects of ovarian hormones in female mice by a peripubertal immune challenge. Horm Behav. 2011;60(4):318–26. Epub 2011/07/05. 10.1016/j.yhbeh.2011.06.005 21722643 PMC3166431

[19] Ismail N, Kumlin AM, Blaustein JD. A pubertal immune challenge alters the antidepressant-like effects of chronic estradiol treatment in inbred and outbred adult female mice. Neuroscience. 2012. Epub 2012/10/06. 10.1016/j.neuroscience.2012.09.047 PMC3567249 23036617

[20] Boulanger LM. Immune proteins in brain development and synaptic plasticity. Neuron. 2009;64(1):93–109. Epub 2009/10/21. 10.1016/j.neuron.2009.09.001 19840552

[21] Carpentier PA, Palmer TD. Immune influence on adult neural stem cell regulation and function. Neuron. 2009;64(1):79–92. Epub 2009/10/21. 10.1016/j.neuron.2009.08.038 19840551 PMC2789107

[22] Deverman BE, Patterson PH. Cytokines and CNS development. Neuron. 2009;64(1):61–78. Epub 2009/10/21. 10.1016/j.neuron.2009.09.002 19840550

[23] Garay PA, McAllister AK. Novel roles for immune molecules in neural development: implications for neurodevelopmental disorders. Front Synaptic Neurosci. 2010;2:136 Epub 2010/01/01. 10.3389/fnsyn.2010.00136 21423522 PMC3059681

[24] Bilbo SD, Biedenkapp JC, Der-Avakian A, Watkins LR, Rudy JW, Maier SF. Neonatal infection-induced memory impairment after lipopolysaccharide in adulthood is prevented via caspase-1 inhibition. J Neurosci. 2005;25(35):8000–9. Epub 2005/09/02. 10.1523/JNEUROSCI.1748-05.2005 16135757 PMC6725459

[25] Bilbo SD, Barrientos RM, Eads AS, Northcutt A, Watkins LR, Rudy JW, et al Early-life infection leads to altered BDNF and IL-1beta mRNA expression in rat hippocampus following learning in adulthood. Brain Behav Immun. 2008;22(4):451–5. Epub 2007/11/13. 10.1016/j.bbi.2007.10.003 17997277

[26] Schwarz JM, Sholar PW, Bilbo SD. Sex differences in microglial colonization of the developing rat brain. J Neurochem. 2012;120(6):948–63. Epub 2011/12/21. 10.1111/j.1471-4159.2011.07630.x 22182318 PMC3296888

[27] Angold A, Costello EJ. Puberty and depression. Child Adolesc Psychiatr Clin N Am. 2006;15(4):919–37, ix Epub 2006/09/06. 10.1016/j.chc.2006.05.013 16952768

[28] Patton GC, Hibbert ME, Carlin J, Shao Q, Rosier M, Caust J, et al Menarche and the onset of depression and anxiety in Victoria, Australia. J Epidemiol Community Health. 1996;50(6):661–6. Epub 1996/12/01. 9039386 10.1136/jech.50.6.661 PMC1060384

[29] Hayward C, Sanborn K. Puberty and the emergence of gender differences in psychopathology. J Adolesc Health. 2002;30(4 Suppl):49–58. Epub 2002/04/12. 11943575 10.1016/s1054-139x(02)00336-1

[30] Bao AM, Swaab DF. Sex differences in the brain, behavior, and neuropsychiatric disorders. Neuroscientist. 2010;16(5):550–65. Epub 2010/10/05. 10.1177/1073858410377005 20889965

[31] Korzhevskii DE, Kirik OV. Brain Microglia and Microglial Markers. Neuroscience and Behavioral Physiology. 2016;46(3):284–90.

[32] Ismail N, Blaustein JD. Pubertal immune challenge blocks the ability of estradiol to enhance performance on cognitive tasks in adult female mice. Psychoneuroendocrinology. 2013;38:1170–7. Epub 2012/12/12. 10.1016/j.psyneuen.2012.11.003 23218519 PMC3604046

[33] Gibb J, Hayley S, Gandhi R, Poulter MO, Anisman H. Synergistic and additive actions of a psychosocial stressor and endotoxin challenge: Circulating and brain cytokines, plasma corticosterone and behavioral changes in mice. Brain Behav Immun. 2008;22(4):573–89. Epub 2008/01/15. 10.1016/j.bbi.2007.12.001 18191534

[34] Gandhi R, Hayley S, Gibb J, Merali Z, Anisman H. Influence of poly I:C on sickness behaviors, plasma cytokines, corticosterone and central monoamine activity: moderation by social stressors. Brain Behav Immun. 2007;21(4):477–89. Epub 2007/02/03. 10.1016/j.bbi.2006.12.005 17267173

[35] Franklin KBJ, Paxinos G. The Mouse Brain in Stereotaxic Coordinates. San Diego: Academic Press; 1997.

[36] Wong A, Sahoo P. A gray-level threshold selection method based on maximun entropy principle. IEEE Transactions on Systems Man and Cybernetics. 1989;19(4):866–71.

[37] Stence N, Waite M, Dailey ME. Dynamics of microglial activation: a confocal time-lapse analysis in hippocampal slices. Glia. 2001;33(3):256–66. 11241743

[38] Chen Z, Jalabi W, Shpargel KB, Farabaugh KT, Dutta R, Yin X, et al Lipopolysaccharide-induced microglial activation and neuroprotection against experimental brain injury is independent of hematogenous TLR4. J Neurosci. 2012;32(34):11706–15. 10.1523/JNEUROSCI.0730-12.2012 22915113 PMC4461442

[39] Carpenter AF, Carpenter PW, Markesbery WR. Morphometric analysis of microglia in Alzheimer's disease. J Neuropathol Exp Neurol. 1993;52(6):601–8. Epub 1993/11/01. 8229079 10.1097/00005072-199311000-00007

[40] Beynon SB, Walker FR. Microglial activation in the injured and healthy brain: what are we really talking about? Practical and theoretical issues associated with the measurement of changes in microglial morphology. Neuroscience. 2012;225:162–71. Epub 2012/07/25. 10.1016/j.neuroscience.2012.07.029 22824429

[41] Verney C, Monier A, Fallet-Bianco C, Gressens P. Early microglial colonization of the human forebrain and possible involvement in periventricular white-matter injury of preterm infants. J Anat. 2010;217(4):436–48. Epub 2010/06/19. 10.1111/j.1469-7580.2010.01245.x 20557401 PMC2992419

[42] Graeber MB. Changing face of microglia. Science. 2010;330(6005):783–8. Epub 2010/11/06. 10.1126/science.1190929 21051630

[43] Schafer DP, Lehrman EK, Stevens B. The "quad-partite" synapse: Microglia-synapse interactions in the developing and mature CNS. Glia. 2012. Epub 2012/07/26. 10.1002/glia.22389 PMC4082974 22829357

[44] Stevens B, Allen NJ, Vazquez LE, Howell GR, Christopherson KS, Nouri N, et al The classical complement cascade mediates CNS synapse elimination. Cell. 2007;131(6):1164–78. Epub 2007/12/18. 10.1016/j.cell.2007.10.036 18083105

[45] Tremblay ME, Stevens B, Sierra A, Wake H, Bessis A, Nimmerjahn A. The role of microglia in the healthy brain. J Neurosci. 2011;31(45):16064–9. Epub 2011/11/11. 10.1523/JNEUROSCI.4158-11.2011 22072657 PMC6633221

[46] Paolicelli RC, Bolasco G, Pagani F, Maggi L, Scianni M, Panzanelli P, et al Synaptic pruning by microglia is necessary for normal brain development. Science. 2011;333(6048):1456–8. Epub 2011/07/23. 10.1126/science.1202529 21778362

[47] Sisk CL, Zehr JL. Pubertal hormones organize the adolescent brain and behavior. Front Neuroendocrinol. 2005;26(3–4):163–74. Epub 2005/11/29. 10.1016/j.yfrne.2005.10.003 16309736

[48] Mohr MA, Sisk CL. Pubertally born neurons and glia are functionally integrated into limbic and hypothalamic circuits of the male Syrian hamster. Proc Natl Acad Sci U S A. 2013;110(12):4792–7. Epub 2013/03/06. 10.1073/pnas.1219443110 23460698 PMC3607016

[49] Ahmed EI, Zehr JL, Schulz KM, Lorenz BH, DonCarlos LL, Sisk CL. Pubertal hormones modulate the addition of new cells to sexually dimorphic brain regions. Nat Neurosci. 2008;11(9):995–7. Epub 2009/01/23. 19160494 10.1038/nn.2178 PMC2772186

[50] Likhtik E, Paz R. Amygdala-prefrontal interactions in (mal)adaptive learning. Trends Neurosci. 2015;38(3):158–66. 10.1016/j.tins.2014.12.007 25583269 PMC4352381

[51] Pfaff DW, Sakuma Y. Deficit in the lordosis reflex of female rats caused by lesions in the ventromedial nucleus of the hypothalamus. J Physiol. 1979;288:203–10. Epub 1979/03/01. 469716 PMC1281422

[52] Pfaff DW, Sakuma Y. Facilitation of the lordosis reflex of female rats from the ventromedial nucleus of the hypothalamus. J Physiol. 1979;288:189–202. Epub 1979/03/01. 469715 PMC1281421

[53] Musatov S, Chen W, Pfaff DW, Kaplitt MG, Ogawa S. RNAi-mediated silencing of estrogen receptor {alpha} in the ventromedial nucleus of hypothalamus abolishes female sexual behaviors. Proc Natl Acad Sci U S A. 2006;103(27):10456–60. 10.1073/pnas.0603045103 16803960 PMC1502479

[54] Davis PG, McEwen BS, Pfaff DW. Localized behavioral effects of tritiated estradiol implants in the ventromedial hypothalamus of female rats. Endocrinology. 1979;104(4):898–903. Epub 1979/04/01. 10.1210/endo-104-4-898 436764

[55] Dewing P, Christensen A, Bondar G, Micevych P. Protein kinase C signaling in the hypothalamic arcuate nucleus regulates sexual receptivity in female rats. Endocrinology. 2008;149(12):5934–42. Epub 2008/07/26. 10.1210/en.2008-0847 18653714 PMC2613064

[56] Doorn KJ, Breve JJ, Drukarch B, Boddeke HW, Huitinga I, Lucassen PJ, et al Brain region-specific gene expression profiles in freshly isolated rat microglia. Front Cell Neurosci. 2015;9:84 10.3389/fncel.2015.00084 25814934 PMC4357261

[57] Soucy G, Boivin G, Labrie F, Rivest S. Estradiol is required for a proper immune response to bacterial and viral pathogens in the female brain. J Immunol. 2005;174(10):6391–8. Epub 2005/05/10. 15879140 10.4049/jimmunol.174.10.6391

[58] Shughrue PJ, Lane MV, Merchenthaler I. Comparative distribution of estrogen receptor-alpha and -beta mRNA in the rat central nervous system. J Comp Neurol. 1997;388(4):507–25. Epub 1997/12/05. 9388012 10.1002/(sici)1096-9861(19971201)388:4<507::aid-cne1>3.0.co;2-6

[59] Rissman EF. Roles of oestrogen receptors alpha and beta in behavioural neuroendocrinology: beyond Yin/Yang. J Neuroendocrinol. 2008;20(6):873–9. 10.1111/j.1365-2826.2008.01738.x 18601711 PMC2667101

